# The emerging outcome of postoperative radiotherapy for stage IIIA(N2) non-small cell lung cancer patients: based on the three-dimensional conformal radiotherapy technique and institutional standard clinical target volume

**DOI:** 10.1186/s12885-015-1326-6

**Published:** 2015-05-02

**Authors:** Wen Feng, Qin Zhang, Xiao-Long Fu, Xu-Wei Cai, Zheng-Fei Zhu, Huan-Jun Yang, Jia-Qing Xiang, Ya-Wei Zhang, Hai-Quan Chen

**Affiliations:** 1 Department of Radiation Oncology, Fudan University Shanghai Cancer Center, 270 Dong An Road, Shanghai, 200032 China; 2 Department of Oncology, Shanghai Medical College, Fudan University, Shanghai, China; 3 Department of Radiation Oncology, Shanghai Chest Hospital, Shanghai Jiao Tong University, Shanghai, China; 4 Department of Thoracic Surgery, Fudan University Shanghai Cancer Center, Shanghai, China

**Keywords:** Non-small cell lung cancer, Survival, Adjuvant radiotherapy, Conformal radiotherapy

## Abstract

**Background:**

The aim of this study was to evaluate the clinical efficacy of postoperative radiotherapy (PORT), administered using three-dimensional conformal radiotherapy (3D-CRT) and our institutional standard clinical target volume (CTV) delineation, for completely resected stage IIIA(N2) non-small cell lung cancer (NSCLC).

**Methods:**

From 2005 to 2012, consecutive patients with pT1-3N2 NSCLC who were treated with PORT employing our institutional CTV delineation after complete surgery or who underwent complete resection in our hospital but without PORT were identified. We excluded patients who had received neoadjuvant chemotherapy or radiation therapy (RT). Kaplan-Meier estimates for locoregional recurrence-free survival (LRFS), distant metastasis-free survival (DMFS) and overall survival (OS) were performed. In the OS estimation, patients who received epidermal growth factor receptor tyrosine kinase inhibitor (EGFR-TKI) during follow-up were censored at the time of TKI initiation.

**Results:**

Data from 70 patients in the PORT group and 287 in the non-PORT group were analysed. All 70 cases received 3D-CRT following our institutional CTV guideline, with a median total dose of 50.4 Gy at 1.8 Gy/fraction. At a median follow-up of 34.3 months for the PORT group and 31.2 months for the non-PORT group, PORT significantly improved local control (5-yr LRFS 91.9% for PORT vs 66.4% for non-PORT, P < 0.001) and OS (5-yr OS 57.5% for PORT vs 35.1% for non-PORT, P = 0.003), whereas no differences in DMFS were noted (P = 0.18). In multivariable analyses, PORT was independently associated with an improved LRFS (HR 0.2, P = 0.001) and OS (HR 0.4, P = 0.001). All patients completed the planned RT dose without interruption of RT due to treatment-related complications.

**Conclusions:**

Our data suggested that PORT administered using the 3D-CRT technique following our institutional CTV delineation guideline resulted in a promising outcome with favourable survival for completely resected IIIA(N2) NSCLC, after controlling for subsequent EGFR-TKI confounding in the OS analysis. Prospective trials are needed to further corroborate these results.

## Background

Completely resected non-small cell lung cancer (NSCLC) patients with pathologically confirmed N2 disease are considered to be a heterogeneous population [1], showing 5-year survival rates ranging from 10% to 30% [2]. Systemic recurrence following surgery is one of the major problems in stage IIIA(N2) patients, and the use of postoperative chemotherapy (POCT) in stage IIIA disease prolongs survival [3]. The value of postoperative radiotherapy (PORT) for completely resected NSCLC remains controversial, as the effect on survival has been inconclusive [4-6]. A meta-analysis of PORT published in 1998 [4] described a relative increase of the risk of death with the addition of PORT for completely resected NSCLC. This detrimental effect was evident among patients who exhibited no mediastinal involvement, whereas in patients with stage III and pN2 disease, a slight increase in survival was detected, although the difference was not statistically significant. Similar results were found when this meta-analysis was updated in 2005 [5]. Recently, several large retrospective studies and a recently published randomized trial have provided evidence of the possible benefit of PORT in completely resected stage IIIA(N2) patients [7-13].

Several limitations of the previous prospective studies included in the PORT meta-analysis have been recognized, including the use of suboptimal radiation techniques and wide irradiation portals. The quality of radiation therapy (RT) was inferior to what is now available, with patients being currently treated using linear accelerators and the three-dimensional conformal radiotherapy (3D-CRT) technique. The irradiation fields employed in most trials have often been large and varying (typically including the entire mediastinum and occasionally the supraclavicular region or contralateral hilum). It has been hypothesized that the toxicity reported in the meta-analysis was related to large field sizes and the use of obsolete radiotherapy techniques [14-16].

Currently, growing evidence suggests that PORT administered using the modern 3D-CRT technique has a favourable effect on the survival of patients with pN2 disease [13,17]. However, there exists significant heterogeneity within the reported studies with respect to the irradiation fields employed for PORT because there is no clear consensus on the definition of the extent of the clinical target volume (CTV) [9-13]. To the best of our knowledge, there is no solid evidence available for the PORT CTV designs used in the currently published prospective trial [13] and ongoing multi-centre phase III studies. Therefore, we designed a patterns-of-failure study after complete surgery in resected pN2 disease to evaluate the rationale of the proposed PORT CTVs based on the most likely sites of nodal failure, and the institutional standard CTV delineation for PORT was developed in our hospital [18].

The aim of the present study was to explore the clinical efficacy of PORT administered using 3D-CRT techniques and the institutional standard CTV delineation guideline in our hospital for patients with completely resected pathologic stage IIIA(N2) NSCLC, in attempt to provide evidence for future phase III clinical trials.

## Methods

### Study population

The study group comprised consecutive patients with completely resected pathologic stage IIIA(N2) NSCLC who were treated with 3D-conformal PORT in accordance with the institutional standard CTV delineation guideline in our hospital between January 2005 and June 2012 (PORT group). During the same period, all consecutive patients with pathologic stage IIIA(N2) NSCLC who had undergone complete resection in our hospital but did not receive PORT were identified retrospectively (non-PORT group). The inclusion criteria for the PORT group and the non-PORT group were the same: complete resection through a surgical procedure of either lobectomy or pneumonectomy; systematic nodal dissection or sampling with a minimum of three N2 stations sampled or completely dissected (one of which must be the subcarinal station) [19]; and histologically proven NSCLC of stage pT1-3N2M0 (according to the TNM classification in the UICC 7th ed. [20]). Complete resection was defined as surgical resection with microscopically tumour-free resection margins (including the bronchial, venous and arterial stumps, peribronchial soft tissue, any peripheral margin near the tumour or additionally resected tissue) and systematic nodal assessment. We excluded patients who died within 4 months of surgery to avoid the influence of perioperative mortality on the study outcomes [7,11]. Patients who received neoadjuvant therapy (chemotherapy and/or RT), showed evidence of metastatic disease, or presented with prior malignancies were excluded. Patients who received adjuvant chemotherapy were included in both of the treatment groups, but the administration of POCT was not mandatory. In addition, patients were routinely assessed through complete clinical and radiological evaluation prior to the initiation of PORT. Patients who exhibited evidence of residual disease, locoregional recurrence and/or distant metastasis prior to PORT were excluded from the PORT group. This study was approved by the Institutional Review Board of Fudan University Shanghai Cancer Center.

### Assessment and definition

The pretreatment evaluation generally included clinical assessment, blood tests, chest computed tomography (CT) scans, bronchoscopy, ultrasound or CT of the abdomen, brain MRI and bone scans. Positron emission tomography (PET)-CT scans were not used as part of the routine preoperative work-up. Patients with mediastinal lymph node enlargement (≥1 cm) in short axis on CT scan were considered as having cN2 lesions.

The patients were generally followed every 3 months after surgery for the first 2 years and every 6–12 months thereafter. Regular follow-up evaluations included clinical assessments, chest CT scans, and ultrasound or CT of the abdomen. Treatment failures were determined by the treating physician based on the available information, including clinical assessments, imaging studies and/or pathology reports. We obtained follow-up information by conducting telephone surveys and by reviewing electronic medical records in the clinic. Disease recurrence at the surgical margin, ipsilateral hilum, and/or mediastinum was considered a local-regional failure (LRF). All other sites of failure, including the supraclavicular zone, contralateral hilum and distant organs, were considered distant metastasis (DM) [21,22]. Data regarding the timing of subsequent epidermal growth factor receptor tyrosine kinase inhibitor (EGFR-TKI) therapy for patients with relapse or progressive disease were recorded.

### Postoperative radiotherapy

All patients in the PORT group were treated using the 3D-CRT technique employing a linear accelerator with 6-MV X-rays. According to our institutional standard PORT CTV delineation guideline, CTVs were delineated separately for left- and right-sided lung cancers [18]. The CTV for left-lung cancers includes the bronchial stump (BS) and lymph node stations (LNS) 2R, 2 L, 4R, 4 L, 5, 6, 7, and 10 to 11 L; and the CTV for right-lung cancers includes the BS and LNS 2R, 4R, 7, and 10 to 11R (according to the 2009 International Association for the Study of Lung Cancer (IASLC) lymph node map [23]). The planning target volume (PTV) was defined as the CTV plus the 0.5-0.8 cm margins. The prescribed total PTV dose was 50.4 Gy, administered daily at 1.8 Gy per fraction, 5 days per week. In the case of cN2 disease or extracapsular node extension, the LNSs with such findings were delineated as CTV-boost; then the 0.5-0.8 cm margin was added to create PTV-boost, and the dose was increased for this volume up to 60.2 Gy. Doses were prescribed to the PTV. The respective 99% PTVs had to be covered by the 95% prescription dose, and 95% PTVs had to be covered by the 100% prescription dose. The dose constraints for the surrounding normal organs were as follows: a maximum dose to the spinal cord of less than 45 Gy; a mean lung dose of less than 15 Gy and less than 25% of the volume of the lung receiving 20 Gy (V20); and a mean heart dose less than 30 Gy.

### Statistical analyses

Comparisons of categorical variables between the groups were carried out using Chi-square test. Locoregional recurrence-free survival (LRFS) was defined from the day of surgery to the day of documented LRF or the last follow-up. Distant metastasis-free survival (DMFS) was defined from the day of surgery to the day of documented DM or the last follow-up. Disease-free survival (DFS) was measured from the day of surgery to disease recurrence, including LRF and DM events, or to the date of death from any cause or the last follow-up. Overall survival (OS) was measured from the day of surgery to the date of death from any cause or the last follow-up. In the OS estimation, patients who received EGFR-TKI for progressive diseases during follow-up were censored at the time of TKI initiation [24,25]. LRFS, DMFS, DFS and OS rates were calculated by the Kaplan-Meier method and compared by means of the log-rank test. Multivariable Cox proportional hazard models (backward conditional stepwise) were used to adjust for differing risk factor distributions between the groups. The statistical analysis was computed using SPSS (version 17.0, SPSS Inc., Chicago, IL). A value of *P* < 0.05 was considered statistically significant.

## Results

### Patient characteristics

Between January 2005 and June 2012, 72 patients with completely resected pT1-3N2 NSCLC who underwent 3D-conformal PORT following our institutional CTV delineation (PORT group) and 303 comparable patients who underwent complete resection in our hospital but did not receive PORT (non-PORT group) were identified using the aforementioned selection criteria. Two patients in the PORT group and 16 patients in the non-PORT group were excluded due to incomplete follow-up data. A total of 357 patients were included in the analysis (Table 1). 30.8% (110/357) of the patients included in the analysis had available PET-CT scans for preoperative staging. Overall, the characteristics of the two groups were comparable with regard to age, clinical N stage, pathologic T stage, tumour location, histology and the involved N2 stations. The application of POCT was relatively well balanced across the two treatment groups; 58 patients (82.9%) in the PORT group and 209 (72.8%) in the non-PORT group received ≥4 cycles of POCT with a platinum-based regimen (P = 0.08). The median numbers of lymph nodes resected in the PORT and non-PORT groups were 16 (range: 3–54) and 20 (range: 5–67), respectively. The median number of N2 stations resected was 4 (range: 3–7) in both analysed groups. The proportions of females and never/light ex-smokers were higher in the PORT group than that in the non-PORT group. More patients with >4 positive lymph nodes or with a lymph node ratio (LNR, defined as the ratio of metastatic to examined lymph node) >20% received PORT. No patients who underwent pneumonectomy received PORT. More patients in the PORT group received subsequent EGFR-TKIs for progressive diseases than in the non-PORT group (P = 0.004).

**Table 1:** **Patient characteristics**

Characteristics	PORT	Non-PORT	*P*-value
	No. (%)	No. (%)	
Patients (N)	70	287	
Age (yr)			0.55
≤60	43 (61.4)	165 (57.5)	
>60	27 (38.6)	122 (42.5)	
Gender			0.03
Male	35 (50)	184 (64.1)	
Female	35 (50)	103 (35.9)	
Smoking history^*^			0.004
Never/light	46 (65.7)	134 (46.7)	
Current/heavy	24 (34.3)	153 (53.3)	
Clinical N status			0.45
cN0,1	35 (50)	158 (55.1)	
cN2	35 (50)	129 (44.9)	
Pathologic T stage			0.05
pT1	15 (21.4)	65 (22.7)	
pT2	53 (75.7)	186 (64.8)	
pT3	2 (2.9)	36 (12.5)	
Type of surgery			0.01
Lobectomy	67 (95.7)	244 (85.0)	
Sleeve lobectomy	3 (4.3)	9 (3.1)	
Pneumonectomy	0	34 (11.9)	
Tumor location			0.13
RUL	26 (37.1)	73 (25.4)	
RML	9 (12.9)	24 (8.4)	
RLL	12 (17.1)	50 (17.4)	
LUL	14 (20.0)	90 (31.4)	
LLL	9 (12.9)	50 (17.4)	
Histology			0.56
Adenocarcinoma	47 (67.2)	169 (58.9)	
Squamous	15 (21.4)	85 (29.6)	
Adenosquamous	6 (8.6)	22 (7.7)	
Large cell	1 (1.4)	9 (3.1)	
Pleomorphic	1 (1.4)	2 (0.7)	
N of positive nodes			0.04
≤4	30 (42.9)	162 (56.4)	
>4	40 (57.1)	125 (43.6)	
LNR			0.002
≤20%	22 (31.4)	150 (52.3)	
>20%	48 (68.6)	137 (47.7)	
Involved N2 stations			0.32
Single	31 (44.3)	146 (50.9)	
Multiple	39 (55.7)	141 (49.1)	
Cycles of POCT			0.08
<4	12 (17.1)	78 (27.2)	
≥4	58 (82.9)	209 (72.8)	
Subsequent EGFR-TKI therapy			0.004
Yes	22 (31.4)	47 (16.4)	
No/unknown	48 (68.6)	240 (83.6)	

Note: *Smoking history was categorized as never/light ex-smokers (<100 cigarettes smoked in the lifetime or smoked ≤10 pack-years, having stopped for ≥15 years) or, the current/heavy ex-smokers.

Abbreviations: LNR = lymph node ratio (defined as the ratio of metastatic to examined lymph nodes), RUL = right upper lobe, RML = right middle lobe, RLL = right lower lobe, LUL = left upper lobe, LLL = left lower lobe, PORT = postoperative radiotherapy, POCT = postoperative chemotherapy, EGFR-TKI = epidermal growth factor receptor tyrosine kinase inhibitor.

### Clinical outcomes

**Figure 1:**
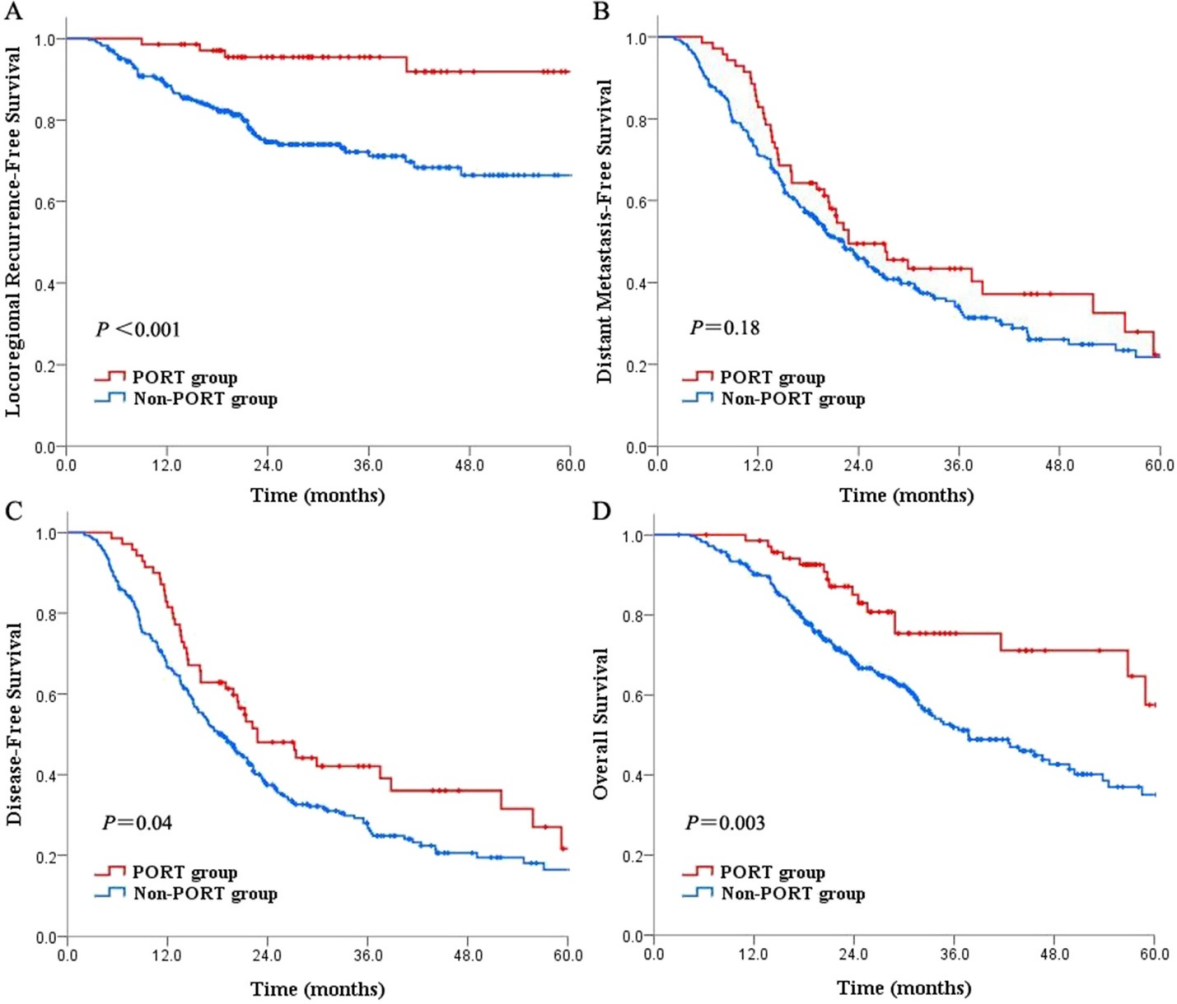
Comparison of **(A)** locoregional recurrence-free survival, **(B)** distant metastasis-free survival, **(C)** disease-free survival, and **(D)** overall survival rates stratified by the PORT and non-PORT groups.

For patients in the PORT and non-PORT groups, the median follow-up times were 34.3 months (range, 17.9-102.6) and 31.2 months (range, 12–101.4) for living patients, respectively. The Kaplan-Meier survival analysis showed that PORT significantly improved locoregional control rates compared with the non-PORT group (1-, 3-, 5-yr LRFS: 98.6%, 95.4%, 91.9% for PORT vs 88.5%, 71.1%, 66.4% for non-PORT, P < 0.001); however, no significant differences in DMFS were noted (5-yr DMFS: 22.3% for PORT vs 21.7% for non-PORT, P = 0.18). The median DFS times were 22.8 months and 18.6 months in the PORT and non-PORT groups, respectively. The results regarding 3- and 5-yr DFS showed a positive trend in the PORT group: 42.1% and 21.6% in the PORT group, respectively, vs 26.8% and 16.4% in the non-PORT group (P = 0.04). The 1-, 3-, and 5-yr OS rates were 98.6%, 75.3% and 57.5%, respectively, in the PORT group, which were significantly higher than the corresponding rates of 90.1%, 51.9% and 35.1% observed in the non-PORT group (P = 0.003) (Figure 1).

Cox multivariate analysis demonstrated that PORT was an independent prognostic factor for improved LRFS (HR: 0.2, 95%CI 0.1-0.5, P = 0.001) and improved OS (HR: 0.4, 95%CI 0.2-0.7, P = 0.001). Smoking history (current/heavy ex-smokers: HR 2.6, 95%CI 1.6-4.4, P < 0.001), cN2 status (HR 1.7, 95%CI 1.0-2.7, P = 0.04) and LNR >20% (HR 2.3, 95%CI 1.4-3.7, P = 0.001) were the other factors independently associated with worse LRFS (Table 2).

**Table 2:** **Univariate and multivariate analyses of factors affecting locoregional recurrence-free survival and overall survival (all patients, N = 357)**

Characteristics	No.	Locoregional recurrence-free survival				Overall survival			
		Univariable		Multivariate		Univariable		Multivariate	
		5-y (%)	*P*	HR (95% CI)	*P*	5-y (%)	*P*	HR (95% CI)	*P*
Age (yr)			0.69		0.99		0.07		0.4
≤60	208	73.3		1		40.0		1	
>60	149	70.0		1.0 (0.6-1.6)		37.6		1.2 (0.8-1.6)	
Gender			<0.001		0.57		<0.001		<0.001
Male	219	64.3		1		32.5		1	
Female	138	83.4		0.8 (0.4-1.7)		50.7		0.5 (0.3-0.7)	
Smoking history			<0.001		<0.001		0.008		0.37
Never/light	180	82.6		1		39.9		1	
Current/heavy	177	60.9		2.6 (1.6-4.4)		36.0		0.8 (0.5-1.3)	
Clinical N status			0.002		0.04		0.003		0.27
cN0,1	193	80.4		1		50.4		1	
cN2	164	61.4		1.7 (1.0-2.7)		26.5		1.2 (0.9-1.7)	
Pathologic T stage			0.11		0.6		0.18		0.77
pT1	80	81.4		1		45.4		1	
pT2	239	70.7		1.0 (0.5-1.9)		39.9		0.9 (0.6-1.4)	
pT3	38	59.1		1.4 (0.6-3.1)		21.8		1.1 (0.6-1.9)	
Type of surgery			0.6		0.26		<0.001		0.24
Lobectomy	323	72.7		1		42.8		1	
Pneumonectomy	34	65.6		0.6 (0.3-1.4)		12.8		1.3 (0.8-2.2)	
Histology			0.01		0.23		0.018		0.05
Non-squamous	257	76.7		1		44.3		1	
Squamous	100	59.4		1.4 (0.8-2.3)		28.1		1.4 (1.0-2.0)	
N of positive nodes			0.002		0.48		<0.001		0.68
≤4	192	80.9		1		53.2		1	
>4	165	60.8		1.3 (0.6-2.7)		21.9		1.1 (0.7-1.9)	
LNR			0.003		0.001		<0.001		<0.001
≤20%	172	81.5		1		55.5		1	
>20%	185	62.7		2.3 (1.4-3.7)		24.7		2.4 (1.7-3.3)	
Involved N2 stations			0.1		0.75		<0.001		0.12
Single	177	76.1		1		56.0		1	
Multiple	180	68.0		0.9 (0.5-1.6)		23.3		1.4 (0.9-2.0)	
Cycles of POCT			0.9		0.79		0.001		<0.001
<4	90	69.5		1		29.8		1	
≥4	267	72.7		1.1 (0.6-1.9)		41.3		0.5 (0.3-0.7)	
PORT			<0.001		0.001		0.003		0.001
No	287	66.4		1		35.1		1	
Yes	70	91.9		0.2 (0.1-0.5)		57.5		0.4 (0.2-0.7)	

Abbreviations: LNR = lymph node ratio, PORT = postoperative radiotherapy, POCT = postoperative chemotherapy, HR = hazard ratio, CI = confidence interval.

### Patterns of first failure

Up to the last follow-up, a total of 248 patients experienced disease recurrence, including 44 (62.9%) in the PORT group and 204 (71.1%) in the non-PORT group (Table 3). Distant metastases represented the most common pattern of failure in both treatment groups. In the PORT group, LRF alone and DM alone occurred in 2.9% (2/70) and 58.6% (41/70) patients, respectively, and 1.4% (1/70) patients exhibited concurrent LRF and DM. In the non-PORT group, 11.1% (32/287) patients exhibited LRF alone; 50.2% (144/287) exhibited DM alone; and 9.8% (28/287) exhibited concurrent LRF and DM. There was a significant reduction in LRF associated with PORT (P = 0.03), but not in the supraclavicular nodes (P = 0.22) or distant metastases (P = 0.21).

**Table 3:** **Patterns of first failure**

Pattern of first recurrence	PORT	Non-PORT	*P*-value
	No. (%)	No. (%)	
All patients (N)	70	287	
Recurrence	44 (62.9)	204 (71.1)	0.18
Local-regional failure alone	2 (2.9)	32 (11.1)	0.03
Local-regional failure & Distant Metastasis	1 (1.4)	28 (9.8)	0.02
Distant Metastasis alone	41 (58.6)	144 (50.2)	0.21
Supraclavicular lymph node alone	4 (5.7)	8 (2.8)	0.22

Abbreviations: PORT = postoperative radiotherapy

### Complications

To date, 143 patients in the non-PORT group have died: 139 (97.2%) from cancer-related causes and 4 (2.8%) from causes unrelated to cancer (cerebrovascular accident in one case, pulmonary infection in one case and coronary artery heart disease in two cases). In the PORT group, 26 patients died, and all of these deaths were cancer-related. All patients completed the planned RT dose without interruption or discontinuation of RT due to treatment-related complications. No other severe late complications were encountered during follow-up.

## Discussion

A growing number of more recent publications have bolstered the use of modern PORT for completely resected stage IIIA(N2) NSCLC [26,27]. However, these reports did not contain detailed information regarding RT, especially concerning the PORT treatment volume. To our knowledge, with the introduction of our institutional standard PORT CTV delineation guideline [18], this is the first report on the clinical efficacy of modern PORT, administered using the 3D-CRT technique and the institutional standard CTV delineation design based on the patterns of local-regional failure data, for completely resected stage IIIA(N2) NSCLC. In the present study, all of the patients included in the PORT group in the analysis were treated using a linear accelerator with the 3D-CRT technique. Moreover, the underlying strength of this study lies in the institutional standard PORT CTV delineation as applied herein [18]. Potential advantages include the following: (*1*) the PORT CTV delineation based on the patterns of failure data might be more reasonable and appropriate; (*2*) this CTV delineation guideline strictly defines LNSs included in the CTV, thus making it more consistent and reproducible in clinical practice; and (*3*) the design of treatment fields tailored to the area most-at-risk for recurrence will reduce the irradiation volume (not including LNSs 1, 3A, 3P, 8, and 9 in our institutional CTV delineation [18]). Thus, it would be of value to assess the efficacy of PORT using the 3D-CRT technique and this institutional standard CTV delineation guideline for completely resected stage IIIA(N2) NSCLC patients.

Prior studies reporting the outcomes of completely resected pN2 patients are outlined in Table 4. We found that the patients treated in both groups analysed in our study (5-yr OS, 57.5% for PORT and 35.1% for non-PORT) yielded superior OS compared with those in previously reported studies [7-13]. The following may be possible explanations for why our survival results in both treatment groups appear to be better than their corresponding historical controls. First, improved survival might be secondary to better patient selection, as a homogeneous group of patients who underwent complete resection of NSCLC and systematic nodal assessment was selected in our study. Second, this difference might be due to the inclusion of a majority of cases receiving adjuvant chemotherapy in our study (all patients in the PORT group and 85% in the non-PORT group received POCT). The ANITA study [8] also demonstrated an advantage of adjuvant chemoradiotherapy in completely resected patients with pN2 disease. It was reported that 5-yr OS was 47.4% under the use of adjuvant chemoradiotherapy, which was relatively comparable to the results of our analysis. Third, the improvement of survival observed in the PORT group likely depends on the application of our institutional CTV delineation guideline, leading to relatively small-sized PORT fields tailored to the area most-at-risk for recurrence after surgery, with good consistency in clinical practice. Miles et al. [14] attempted to estimate the field size dependence of RT-induced mortality and tumour control in the postoperative setting. It has been shown that RT-induced mortality is strongly dependent on the field size, which may partly offset the OS benefit afforded by PORT. The incongruity between an improvement in local control and a decrease in survival may have been secondary to RT-induced complications.

**Table 4:** **Rates of overall survival and locoregional recurrence rates after complete resection in pN2 NSCLC**

Author	Year	Stage	No. of patients	5-y OS (%)		5-y LRR rates (%)	
				S	S + PORT	S	S + PORT
SEER [7]	2006	pN2	1987	20	27	NS	
ANITA [8]	2008	pN2	106(observation)	16.6	21.3	42.1^*^	22.1^*^
			118(chemotherapy)	34	47.4	25.7^*^	14.6^*^
Zou et al. [9]	2010	pN2	183	22.2	30.5	66	27
Scotti et al. [10]	2010	pN2	175	NS		44	20
Dai et al. [11]	2011	IIIA-N2	221	30.6	36.6	53	36
Mantovani et al. [12]	2013	pN2	66	NS	37	NS	28
Shen et al. [13]	2014	IIIA-N2	135	27.5	37.9	49.3^*^	27.3^*^

Note: *indicates crude LRR rates. Abbreviations: NS = not stated, OS = overall survival, LRR = locoregional recurrence, S = surgery, S + PORT = surgery plus postoperative radiotherapy, SEER = Surveillance, Epidemiology, and End Results, ANITA = Adjuvant Navelbine International Trialist Association.

Our results showed that the locoregional recurrence (LRR) rate was reduced from 33.6% to 8.1% with the administration of PORT (P < 0.001), and 5-yr OS for patients who received PORT was 57.5%, which was obviously higher than in patients not receiving PORT (5-yr OS, 35.1%). A similar reduction in LRR rates with a survival benefit was reported by previous retrospective series [7-9,11]. Our findings are also congruent with a recent meta-analysis study [17] echoing a similar increase in local control and OS for completely resected stage IIIA(N2) NSCLC. In this meta-analysis, it was reported that the application of PORT using modern techniques was estimated to reduce the LRF rate to 10% and increase absolute 5-year OS by 13% [17].

The type of disease failure pattern predominated by DM is also quite similar to the results reported in other trials [9-12]. The patterns of failure outcomes after surgery with or without PORT reported herein are in keeping with the clinical efficacy of PORT as well, demonstrating that PORT is able to reduce locoregional recurrences, but not in supraclavicular nodes or distant metastases. Of note, distant metastases remain more frequent in completely resected pIIIA(N2) disease, despite the addition of PORT, thereby encouraging further exploration. It is possible that patients with NSCLC have occult systemic disease, especially in pN2 stages, and that PORT alone is not adequate to confer a survival benefit without effective systemic control by POCT. In the light of our data, it can be concluded that the major problem for this patient population remains the high risk of distant metastases, indicating the necessity for the development of optimal adjuvant or systemic treatment strategies.

The current study is observational in nature and as a result cannot prove a direct causal relationship between PORT and prolonged survival. However, this link is highly plausible for the following three major reasons. First, it was demonstrated that PORT was independently associated with improved OS according to the multivariate analysis. Second, although the baseline data were not balanced in the two treatment groups, these baseline imbalances might bias our results towards either the PORT or non-PORT group. In our study group, patients whose tumour characteristics (>4 positive lymph nodes and LNR >20%) were perceived to be worse might have been referred for PORT more often. Third, the fact that EGFR-TKI therapy subsequently administered for relapse or progressive disease might obscure improved survival should be considered and accounted for in the evaluation of OS endpoints in current clinical practice. Thus, we attempted to control for the disparity in subsequent EGFR-TKI therapy between the two treatment groups by applying a censoring approach at the OS estimation [24,25]. This is one of the main differences between our study and most other studies, including a recently published small randomized trial conducted in China [13]. In consideration of these factors, the application of PORT using the 3D-CRT technique and our institutional standard CTV delineation guideline might confer a significant survival advantage for completely resected stage IIIA(N2) patients based on our present descriptive data.

In the present study, several clinical parameters (current/heavy ex-smoker, cN2 status and LNR >20%) were identified as indicators of a high risk of LRF after complete surgery in resected pN2 patients. These findings of this study are in line with the results of previous studies on the prognosis of completely resected IIIA(N2) patients [1,28-30]. The identification of high-risk prognostic factors for LRF after complete surgery could be applied to individualized clinical decision making (as completely resected patients with pN2 disease can have different prognoses) and in stratifying the randomization applied in clinical trials.

We acknowledge that there are some limitations inherent to this retrospective study, such as selection bias, missing data and inconsistent follow-up intervals. Comparisons between the PORT and non-PORT groups have been hampered by the retrospective nature of the study and difficulty in controlling for confounding variables. The two populations were not well-balanced with respect to several clinicopathologic factors. In fact, there existed both favourable and adverse prognostic confounders that may have biased the results towards either the PORT or non-PORT group. Furthermore, we could not differentiate which factors among these potential confounders presented larger values and significantly contributed to the outcomes presented herein. Another important limitation was that the subsequent EGFR-TKI therapy administered for relapse or progressive disease after complete surgery was not strictly controlled for and was not well-balanced in the two groups. It was noted that more patients in the PORT group received EGFR-TKI than in the non-PORT group, which might result in a bias towards improved survival results. However, we have taken appropriate steps, including statistical considerations (censoring the analysis at the time of TKI initiation in the OS estimation), in an attempt to control for the potential impact of this disparity on OS. Finally, our study is limited by the relatively small number of patients analysed in the PORT group. Therefore, there is still a need for a prospective study to validate the efficacy of 3D-conformal PORT in accordance with our institutional standard CTV delineation guideline.

## Conclusions

Our data suggested that PORT administered using the 3D-CRT technique following our institutional standard CTV delineation guideline resulted in promising outcomes regarding local control and survival improvements for completely resected stage IIIA(N2) NSCLC patients, after controlling for the confounding effect of subsequent EGFR-TKI therapy in the OS analysis. Prospective and comprehensive trials are needed to further corroborate these results. This report may lay the groundwork for future phase III clinical trials of 3D-conformal PORT following the standard CTV delineation guideline.

### Consent

Written informed consent was obtained from the patient for the publication of this report and any accompanying images.
